# Kinship underlies costly cooperation in Mosuo villages

**DOI:** 10.1098/rsos.171535

**Published:** 2018-02-21

**Authors:** Matthew Gwynfryn Thomas, Ting Ji, Jiajia Wu, QiaoQiao He, Yi Tao, Ruth Mace

**Affiliations:** 1 Department of Anthropology, University College London, 14 Taviton Street, London WC1H 0BW, UK; 2 Key Laboratory of Animal Ecology and Conservation Biology, Centre for Computational and Evolutionary Biology, Institute of Zoology, Chinese Academy of Sciences, Beijing 100101, People's Republic of China; 3 Life Sciences, Lanzhou University, 222 Tianshui South Road, Lanzhou, Gansu Province, 730000, People's Republic of China

**Keywords:** reciprocal altruism, farmers, kin selection, China

## Abstract

The relative importance of social evolution theories such as kin selection, direct reciprocity and need-based transfers in explaining real-world cooperation is the source of much debate. Previous field studies of cooperation in human communities have revealed variability in the extent to which each of these theories explains human sociality in different contexts. We conducted multivariate social network analyses predicting costly cooperation—labouring on another household's farm—in 128 082 dyads of Mosuo farming households in southwest China. Through information-theoretic model selection, we tested the roles played by genealogical relatedness, affinal relationships (including reproductive partners), reciprocity, relative need, wealth, household size, spatial proximity and gift-giving in an economic game. The best-fitting model included all factors, along with interactions between relatedness and (i) reciprocity, (ii) need, (iii) the presence of own children in another household and (iv) proximity. Our results show how a real-world form of cooperation was driven by kinship. Households tended to help kin in need (but not needy non-kin) and travel further to help spatially distant relatives. Households were more likely to establish reciprocal relationships with distant relatives and non-kin but closer kin cooperated regardless of reciprocity. These patterns of kin-driven cooperation show the importance of inclusive fitness in understanding human social behaviour.

## 1. Introduction

Humans are highly social, and live in groups that cooperate extensively with interdependent kin and non-kin. The variability and flexibility observed in cooperative strategies across communities of humans throughout the world has led to a profusion of explanations for the evolution and maintenance of our extreme social behaviour: some derived from models applicable to a wide variety of organisms; others specific to us.

The inclusive fitness framework posits that social behaviour will be selected for when the indirect fitness benefits, tempered by the proportion of shared genes, outweigh the direct fitness costs of that behaviour (known as ‘Hamilton's rule’: rb>c [1]). Typically, this can come about with the aid of mechanisms—such as kin recognition and limited dispersal—that allow for assortment among genetic relatives [2]. While such assortment can select for cooperation, under some circumstances such as shared resources or limited mates, genetic relatives might also compete [3].

The importance of inclusive fitness explanations for our large-scale cooperation has been debated, with some researchers emphasizing other evolutionary mechanisms such as direct reciprocity, need-based transfers (NBTs), reputation-based partner choice, signalling and cultural group selection, over and above kinship [4–8]. Here, we will investigate the roles played by relatedness, reciprocity and NBTs in structuring decisions to engage in a costly form of cooperation for members of a farming community in southwest China. (In this contribution, we define cooperation as a social behaviour that benefits the fitness of another individual, without necessarily proving detrimental to the cooperator's fitness [9].)

Beyond cooperation driven by shared ancestry, humans also recognize and work with extensive networks of non-kin who are considered as family [10], including affines (spouses and in-laws) and fictive kin (e.g. ‘brothers from another mother’, fatherlands and sisterhoods). Affinal kin, in particular, might cooperate due to aligned reproductive interests in descendants [11]. Extending the status of ‘family’ to friends and others expands social networks, forging deep ties of mutual obligation, ameliorating social isolation and perhaps substituting for absent biological family [12–15].

Direct reciprocity (also known as reciprocal altruism [16]) is another evolutionary mechanism that can promote cooperative strategies in populations. The canonical reciprocal strategies are tit-for-tat (cooperate first, then copy your social partner's actions) and win-stay, lose-shift (repeat the same action if successful, otherwise change action) operating in an iterated prisoner's dilemma situation [17,18]. For human and non-human primates, at least, reciprocity can involve exchanges in the same commodity or trade across domains of cooperation [19,20].

In contrast to forms of direct reciprocity that require an actor to track and respond to their history of interactions with social partners, NBTs do not require memory or cognitive load. NBTs are initiated by the (potential) recipient of a cooperative action; such transfers are free of obligations to repay (either in-kind, entirely or even at all) and thus do not create debt bondage between social partners [21]. Theoretically, NBTs allow risk-pooling, increase survival and decrease wealth inequality [22,23].

The theories of cooperation outlined above are not mutually exclusive. A meta-analysis of direct reciprocity, kin selection and tolerated scrounging^1^ in 32 primate populations (including human forager groups) found that, overall, reciprocity was the strongest predictor of food sharing, although its effect was highly variable between societies and statistically indistinguishable from the positive effect of kinship [20]. Similarly, a meta-analysis of non-human primate grooming behaviour found that reciprocity was the relatively stronger predictor of cooperation compared to kin selection, although both evolutionary mechanisms positively predicted grooming [24].

For humans, reciprocal partnerships can also emerge from the interplay between cultural norms of widespread sharing, kinship relations and constraints (such as spatial distance between households). A study of food sharing in Namibia, for example, found that specific reciprocal partnerships were spurred on by differences in social partners (e.g. quality, kinship and proximity) that people had access to via a norm of unconditional giving [25].

Field studies of cooperation in human societies have uncovered the sheer flexibility characteristic of our species. Households cooperate in many ways, from farming, hunting and sharing food, through to policing their communities and arranging marriages. Closer kin are often the preferred recipients of help [26–31], especially relatives who are in need [32,33]. Indeed, several studies have shown the importance of inclusive fitness in explaining costly forms of cooperation [33–37]. Kinship is not always an important factor in cooperation [38,39], however, and might be more important for behaviour such as childcare rather than labouring or food sharing in some societies [19]. Therefore, we derive several mutually inclusive predictions about cooperation among households in our study area (table 1). Given the importance of kinship for cooperation in human communities, we predict that households will be more likely to help households in which closer kin live (H1) and that people will travel further to help closer kin (H2). In addition, people will be more likely to help kin who are relatively more needy (H3).

**Table 1.** Predictions tested in this study. See main text for context.

hypothesis	supported?
H1. Households will be more likely to help on a farm where closer kin live	yes
H2. People will travel further to help closer kin	yes
H3. People will be more likely to help kin who are relatively needier (measured as producer : consumer ratios)	no
H4. Households will engage in directly reciprocal relationships regardless of kinship	no
H5. Households will be more likely to help relatively less wealthy households	no

Reciprocal relationships between households might mitigate the risks of living in variable environments [40]. Reciprocity can be the organizing principle for food transfers [32] and cooperative hunting [41], among other domains of helping [27]. Labour exchange, such as through working on the farms of other households—the form of cooperation we study here—might help create economies of scale, where investment costs are reduced while output increases [42]. We thus expect that households in our study villages will be more likely to engage in directly reciprocal relationships, even without kinship (H4; table 1).

Households with relatively more consumers also tend to receive more help [26,27], although this is not a universal pattern [40]. Regardless, larger, wealthier and/or more productive households might be more likely to provide goods and services to others [40,43]. Therefore, we predict that households in our study area will be more likely to help relatively less wealthy households (H5; table 1).

We extend the field studies reviewed above by investigating the evolutionarily salient factors predicting a time-consuming and energetically costly form of cooperation (labouring on another household's farm). We also explicitly compare this real-world, costly cooperation to cost-free allocation decisions in a gift game, as used in other studies of cooperation [29,44,45]. This paper will compare the relative importance of four evolutionary mechanisms of cooperation—relatedness (genealogical and affinal), reciprocity, need-based helping and rewards—in predicting costly cooperation in a farming community from southwest China.

## 2. Material and methods

### 2.1. Study area

‘Mosuo’ refers to an ethnic group in rural southwest China, located around Lugu Lake on the border of Sichuan and Yunnan provinces. Mosuo (also known as Na) social life is typically organized around matrilineal households in which family members spend most if not all their lives (duolocal residence) [46].

Agriculture is a primary means of subsistence in this area. Members of different households come together to help one another during planting and harvesting seasons; everybody works in the fields during this time, regardless of gender or age. Households also cooperate in the construction of new houses, share funeral costs if the deceased's household cannot afford the ceremony, and jointly invest in economic ventures [47].

The area has become an increasingly popular tourist spot, which has led to a number of Mosuo households deviating from matrilineal norms due to a mixture of cultural diffusion and economic motivations [48]. Many households on the Sichuan province side of the lake, the site of this study, follow the duolocal Mosuo way of life, although tourism and more intermarriage with Han people are causing this to shift [47,49].

Within the matrilineal families, all residents share the fruits of household labour. Sisters reproduce communally. Older sisters invest more time in farm work and have correspondingly higher reproductive success compared to their younger sisters [50]. (Note that rural ethnic minorities like the Mosuo people have been allowed 2–3 children since the 1979 fertility policy.)

In traditional Mosuo life, ‘marriage is rejected as an institution that disrupts household harmony’ [51, p. 487]. Harmonious relationships are the ideal between household members [47] and grandmothers tend to be the heads of houses. Family members will eat and farm together, pool money and care for children communally. Big families are preferred but households can fission if they become too large or if relationships are riven by conflict [48]; however, fissions are seen as shameful and to be avoided [52].

One aspect of Mosuo culture is *sese* or *zohoun* (walking marriage). A man in a walking marriage will visit his partner's house during the night and return to his natal household at daybreak. Once a union is publicly recognized, the male may eat with or give gifts to his partner's family [47]. Since the 1980s, formal marriage has been a requirement for reproduction for most people throughout China as part of the government's family planning policy, so the idea of conjugal partners has become more similar to the Han norm if a child is involved, even when partners live apart. For simplicity, we use the terms ‘husband’ and ‘wife’ to refer to male and female *zohoun* and/or marriage partners identified as such in the household surveys.

### 2.2. Data collection

All data collection was carried out by Jiajia Wu, Qiao-Qiao He and Ting Ji. Demographic surveys were conducted in five villages in Sichuan province around Lugu Lake during 2012. One adult was interviewed on behalf of all household members about details including name, age, sex, ethnic group, names of spouses and parents. GPS locations were also captured for households. Pedigrees were created by linking every person in the census to their mother and father.

In 2013, individuals were gathered together in groups in several public locations in the villages, to have the games explained to them, and then the actual games were played one by one in private in a nearby room. Participants played a gift game in which they gave gifts to individuals anywhere in the study villages. Participants were endowed with 15 yuan, which they could give—in five yuan denominations—to between one and three recipients; players were not allowed to keep any of their endowments for themselves. We aggregated gift-giving at the household level.

Data on working in the fields was collected by moving from farm to farm during harvest or planting season and recording the composition of workers on each farm. Spot observations of all those people working on each farm (defined as those present on the field when we arrived) were conducted during the planting seasons of 2011 and 2012 and the harvest season of 2012. Locations were randomly sampled within the study villages, giving unbiased, although incomplete, coverage. Workers' names were collected and the names were linked to their records in our demographic database.

Wealth ranking of households was conducted by 1–3 senior people in each village. The fieldworkers presented them with cards with the names of the heads of each household; they then divided the cards into three piles: rich, medium and poor. The villagers further divided ‘medium’ into another four piles, leaving a total of six piles of households: very rich (1) to very poor (6). The people who did the wealth ranking were usually heads of that village who were familiar with every household. Note that wealth ranks can only be interpreted within the context of each village—e.g. a household ranked 3 in village A does not necessarily have equivalent wealth to a household ranked 3 in village B.

### 2.3. Data preparation

Relatedness between each pair of individuals was calculated from the pedigree data using a modified version of PyPedal [53]. Relatedness between households was calculated as the mean relatedness between each pair of individuals in the ego and alter households [28,32,54]. Note that we do not know levels of paternity certainty but it seems not to be especially high: 20% of women had reported offspring by more than one partner. Affinal networks were constructed based on the names and households of individuals' spouses and aggregated at the household level.

Distance in kilometres between households—a proxy for distance travelled to help, because fields are normally fairly close to houses—within the same village was calculated from the longitude and latitude GPS coordinates. Household size was calculated as the total number of people living there at the time of the census. The three seasons of farm observations were aggregated by household.

Relative wealth ranking was calculated by subtracting ego's rank from alter's rank. Positive relative rank means the household receiving help (alter) was poorer than the helping house (i.e. alter's wealth rank > ego's rank). Larger differences in rank indicate greater wealth disparity.

Relative need was defined as the ratio of consumers to producers in a household [27,40]. ‘Consumers’ represents dependents: defined in this case as the number of children younger than the lowest recorded age at first birth in the population (15 years). Larger values of relative need means that there were more dependent children per adult in a household.

Household dyads were limited to those occurring within the same village and include only those headed by Mosuo people. Binary variables were coded as 1 for each household dyad if any member of one household:

— was ever observed helping on the farm of another household (the response variable in our analyses);
— gave a gift to any member of another household in the gift game;
— had any children (of any age, not just less than 15 years) in another household; and
— had a partner (somebody with whom they reproduced) residing in another household.

### 2.4. Statistical analysis

We fitted generalized estimating equations (GEEs) to investigate the importance of relatedness, reciprocity, need and gifts in predicting whether any residents of a household (ego) ever worked on an alter household's farm. GEEs were specified with an exchangeable correlation matrix and observations were clustered on the ego households. We used model selection on a candidate set of models representing the hypotheses outlined in the Introduction (table 4). To select the best models, we compared the quasi-likelihood under the independence model information criteria [55].

In order to allow comparison of coefficients within models, we standardized continuous parameter estimates over 2 s.d. and mean-centred binary estimates [56]. Parameter estimates reported in the main text are unstandardized log odds unless otherwise stated.

All analyses were conducted in R v. 3.3 [57].

## 3. Results

**Figure 1.**
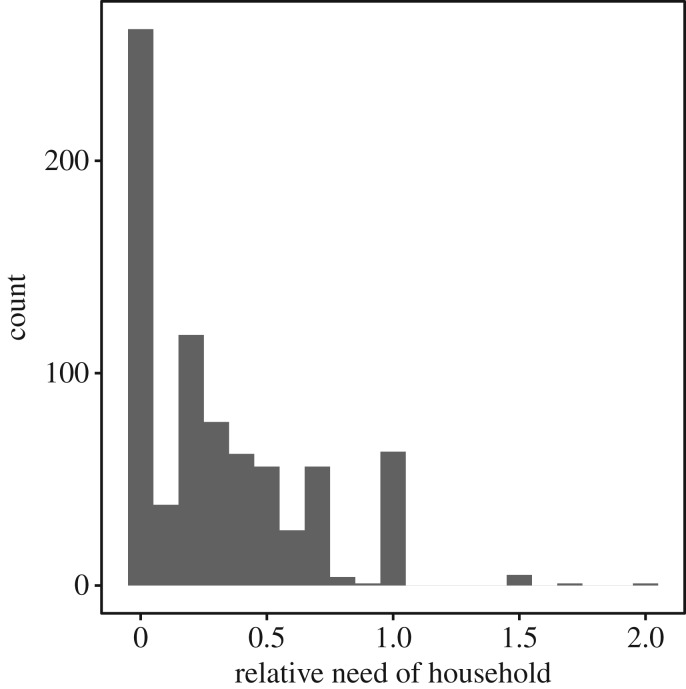
Histogram of relative need in each household (number of dependent children : number of adults). There were seven households containing more children less than 15 years than adults (relative need greater than 1). Relative need of zero means the number of dependent children and the number of adults is balanced.

**Figure 2.**
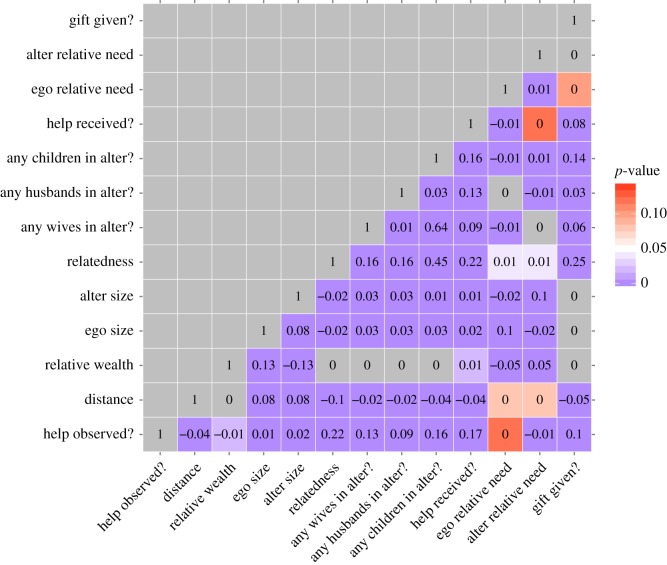
Bivariate correlations between predictor variables and the response variable (help observed). Numbers within the cells are Pearson's correlation coefficients. Blue cells are statistically significant (*p *< 0.05), with darker shades as *p* approaches zero; white cells are borderline statistically significant; red and grey cells are not statistically significant.

There were 770 households across the five study villages, forming 128 082 dyads (table 2). The number of dyads was derived from summing the total number of within-village dyads: n × (n−1), where *n* is the number of houses in a village. Note this totals 128 824 before excluding 742 dyads with missing data. Table 3 and figure 1 show the distributions of between-household relatedness and within-household relative need; figure 2 shows correlations between variables).

**Table 2.** Descriptive statistics, stratified by village. ‘Distance’ refers to kilometres between households; ‘modal gifts’ and ‘modal help’ refer to the most common number of gifts given between households and most common number of times a member from one household was observed helping on another's farm. Note that the median number of gifts and amount of help given are also 1 for all villages.

village	no. houses	mean HH size	s.d. HH size	mean distance (km)	s.d. distance (km)	modal gifts	modal help
A	120	7.000	3.007	0.996	0.694	1	1
B	244	6.918	2.943	1.348	0.999	1	1
C	131	6.969	2.572	1.438	1.027	1	1
D	119	5.580	1.839	0.673	0.432	1	1
E	156	5.045	1.776	0.705	0.540	1	1

**Table 3.** Breakdown of the number of dyads related at different intervals. Here we only count each ego–alter pair once so the counts sum to 64 041 rather than 128 082.

relatedness	no. dyads
[0,0.0039)	60 227
[0.0039,0.0078)	542
[0.0078,0.015)	622
[0.015,0.031)	751
[0.031,0.063)	914
[0.063,0.125)	566
[0.125,0.25)	388
[0.25,0.5)	31

**Figure 3.**
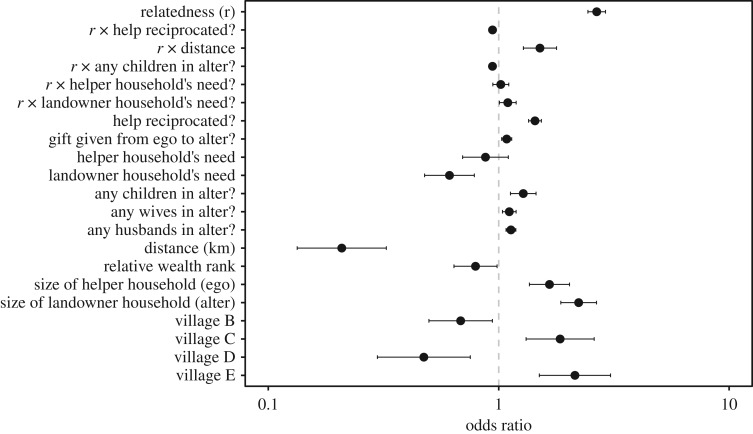
Odds ratios from the best-fitting generalized estimating equation predicting farm labour (table 4). Estimates were standardized over 2 s.d. to allow comparison between continuous and binary predictors [56]. Error bars are 95% confidence intervals. Intercept (OR = 0.002 [0.001, 0.002]) not shown for clarity.

The best-fitting model predicting help on another household's farm contained all four mechanisms of cooperation: relatedness; reciprocated help; relative need; and gifts (table 4). For ease of comparing effect sizes for continuous and binary predictors within the best-fitting model, we present odds ratios with 95% confidence intervals standardized over 2 s.d. in figure 3 (see Materials and methods); for ease of real-world interpretation, we present unstandardized log odds in the main text.

**Table 4.** Candidate set of generalized estimating equations (GEEs) predicting farm labour in dyads of households (ego–alter pairs) within villages. All models except the intercept-only one controlled for distance between ego and alter households, the number of people living in each household, and their relative wealth rank. ‘Relatedness’ models also include terms for relatedness × distance between households, the presence of partners and children in alter households, and an interaction between relatedness and the presence of children in alter households. See Material and methods for details about the operationalization of other predictors. The best-fitting model (bold) is analysed in the main text.

model	log-likelihood	*Δ*QIC	weight
**relatedness × relative need + relatedness × reciprocity + gifts**	−**2593**.**817**	**0**.**000**	**1**
relatedness × relative need + reciprocity + gifts	−2618.853	47.498	0
relatedness + reciprocity + relative need + gifts	−2620.735	47.627	0
relatedness + reciprocity + relative need	−2627.013	57.873	0
relatedness + reciprocity	−2632.840	64.050	0
relatedness + relative need + gifts	−2664.148	125.778	0
relatedness + gifts	−2670.361	133.036	0
relatedness + relative need	−2671.040	137.701	0
relatedness	−2676.891	144.163	0
reciprocated help	−3103.617	978.718	0
gifts	−3222.550	1214.000	0
relative need	−3306.860	1381.411	0
control model (distance + HH size + relative wealth)	−3314.098	1392.992	0
intercept-only	−3557.519	1865.631	0

**Figure 4.**
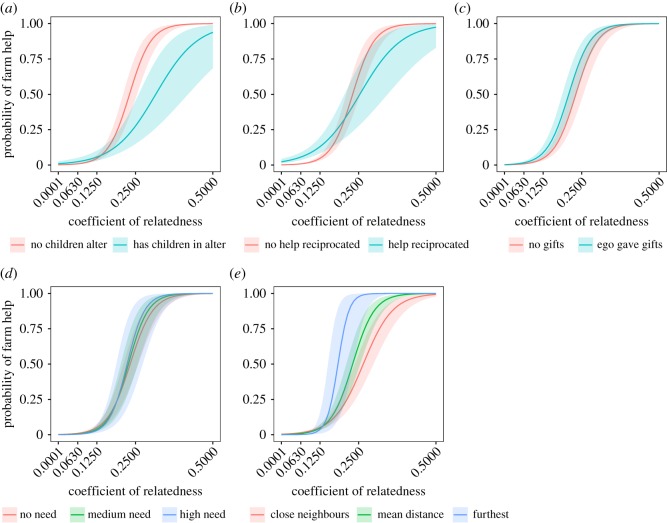
Predicted probabilities of farm labour for household dyads within the same village. All panels show the combined effect of relatedness between households (*x* axes) and (*a*) whether or not the helping (ego) household has any children present in alter; (*b*) whether or not alter helped on ego's farm; (*c*) whether or not ego gave alter gifts; (*d*) alter's relative need (no need = 0, medium need = 1, high need = 2); (*e*) spatial distance between ego and alter (for close neighbours, distance = 0 km; mean distance = 1.130 km; furthest = 5.310 km). All other predictors were set to the population mean values.

Closer relatedness between households strongly predicted an increased likelihood of farm help (log odds = 19.528; 95% CI [16.708, 22.348]; figures 3 and 4), supporting H1. Households were more likely to help one another if children (of any age, not just less than 15 years) of any member of one household resided in the other (log odds = 1.905; 95% CI [0.918, 2.891]). However, the negative interaction between child presence and between-household relatedness (log odds = −13.689; 95% CI [−19.029, −8.349]) means that help was less likely as relatedness increased when children lived in the landowning households (figure 4*a*). Therefore, farm labour might act as a form of parental investment when children do not live with other people who are related to the helper(s).

Help was given to closer neighbours (i.e. distance had a strong negative effect: log odds = −0.886; 95% CI [−1.136, −0.636]). The interaction between distance and relatedness had a positive effect (log odds = 6.598; 95% CI [3.95, 9.246]), meaning the people were willing to travel further to help relatives, supporting H2 (figure 4*e*).

Households with a higher relative need—that is, a higher ratio of consumers (children under 15 years) to producers—were not less likely to give help (log odds = −0.206; 95% CI [−0.561, 0.149]) but were less likely to receive help (log odds = −0.767; 95% CI [−1.153, −0.38]). Although the interactions between relatedness and relative need appeared in the best model, it had a small, uncertain effect on the probability of farm labour and did not support H3 (figure 4*d*). Households were more likely to labour on the farms of households that reciprocated the help, unless they were closely related, opposing H4 (figure 4*b*). Households were slightly more likely to help on the farms of households they had given gifts to (log odds = 0.649; 95% CI [0.241, 1.058]), although the effect is small (figure 4*c*).

Farm help was more likely if there was at least one female reproductive partner (log odds = 1.18; 95% CI [0.417, 1.943]) or male reproductive partner (log odds = 1.367; 95% CI [0.823, 1.911]) present in the alter household. Larger households were more likely to receive help (log odds = 0.147; 95% CI [0.114, 0.179]) and give help (log odds = 0.093; 95% CI [0.056, 0.13]). Increasing relative wealth rank was associated with a lower probability of helping (log odds = −0.056; 95% CI [−0.107, −0.004]), meaning that wealthier households were less likely to help poorer households (positive relative rank), not supporting H5, whereas poorer households were more likely to help richer households (negative relative rank).

## 4. Discussion

Our results show the relative importance of genealogical kin for structuring a costly measure of real-world cooperation—labouring on farms—compared to other evolutionarily important mechanisms of cooperation, such as reciprocity and NBTs. Kinship was the strongest positive predictor of farm labour in the study villages. Farm help was likely to have been reciprocated, except if the help was provided by closely related households; closer kin cooperate regardless of reciprocity.

Affinal relationships were also positive predictors of cooperation, as observed elsewhere [27,35]: people were more likely to help on the farms of households containing their sexual partners. The presence of children in another household was associated with a higher likelihood of helping on that household's farm. (Note that we defined ‘children’ in this case as offspring of any age, not just dependents.)

Cooperation was somewhat constrained by spatial distance, meaning that households were more likely to help their neighbours: a pattern observed in other societies [19,25,27,28]. However, people were more likely to travel further to help relatives. Contrary to predictions, poorer households were not more likely to receive help. Similarly, households with greater need (i.e. a higher proportion of consumers to producers) were less likely to receive help on their farms, except from kin.

Gift-giving in an economic game positively predicted observed farm labour. Allocation decisions in gift games have become popular as a simple proxy of cooperation or to reveal underlying social relationships [29,44,45]. By comparing game behaviour to observations of a salient and costly form of cooperation, our results suggest that this form of economic game has external validity and can be an appropriate measure of cooperation.

Labouring on another household's farm—the costly form of cooperation investigated here—could potentially act as a form of investment in future reproduction. Labour exchange might also occur due to ‘competitive altruism’, where individuals who are better cooperators, or have the reputation of being a better cooperative partner, become preferred social or reproductive partners [58]. Increased helping for richer households may be an immediate response to being well fed while helping, or to keeping on good terms with more affluent and influential families, including for seeking future mates or allies. A study of labour exchange in Dominica found that men working more often had greater reputations for altruism and were, in turn, chosen more often as cooperative partners, although their reputation was not associated with mating success [59]. People living in the same hamlets tended to exchange labour with one another, suggesting a similar spatially constrained pattern of cooperation as in this study.

The lack of helping for those in need is in contrast with some findings from pastoralist societies [23] and from hunter–gatherers [60]. It may be that poverty in farming communities is more strongly related to long-term variables such as the size or quality of land owned, which is less likely to fluctuate in the way that pastoralists' herds or foragers' needs can do in response to ecological uncertainty. Therefore, poverty in a farmer may be more persistent and not of the type that will generate reciprocal altruism (which is predicted to be more likely when both poor and rich live in fear that they may one day be the one that is in need). Alternatively, the duolocal residence system, which we have shown elsewhere to be associated with relatively low levels of between-household cooperation compared to other residence systems in the region [61], may simply reduce between-household cooperation. Formal affinal links with other households can be weaker than in groups where one sex disperses.

This paper only investigated one kind of non-kin relationship: mating partners; future work could begin to explore the importance of other non-kin relationships such as ritual partners and friendships [35,62,63]. We have shown in a companion paper [64] that a reputation for supposedly being a ‘poison giver’, predicts less help from those not so labelled, but more help from those that share this harmful label. Reputation in some domains is clearly important in structuring patterns of help. However, we showed that that particular tag does not correlate with cooperativeness in an economic game [64], so its origins and function are unclear. Furthermore, the cultural tag correlated slightly positively with wealth, not negatively, so assortment on the tag is not likely to explain why those apparently in most need got less help.

This study has a few limitations. We conducted correlational analyses of cross-sectional and sparse data on farm help and behaviour in an economic game. Future work should investigate the longitudinal dynamics of cooperation, especially the processes underlying the formation of reciprocal helping relationships and other ties in Mosuo social networks. It would also be interesting to examine if and how cooperation changes in response to changes in household need over time (e.g. as dependent children grow up and become producers), as well as the links between individual differences in cooperative behaviour rather than just cooperation aggregated at the household level. Future studies could also explore cooperation across domains other than labour, such as childcare or food production [19].

Theoretical models of kin selection generalize the concept of relatedness beyond shared ancestry (pedigree or genealogical relatedness) to include all forms of assortment on genotype [65]. This redefines relatedness as a statistical concept capturing the idea that any two individuals might be less or more related to one another than either is to their local group, potentially allowing for negative values of relatedness [66]. Empirical studies, on the other hand, tend to operationalize relatedness as Wright's coefficient, derived from genealogical data. One potential avenue for future conciliation between these approaches could be to take advantage of ever-cheaper DNA sequencing technology to empirically test for differences in the explanatory power of genealogical relatives and genetic relatives without shared ancestry in predicting cooperative behaviour and reproductive success.

Our work supports the idea that there appears to be something ‘special’ about relatedness through shared ancestry compared to sharing alleles at particular loci for other reasons. Identity-by-descent can be important because genealogical relatives are equally related across the whole genome (more or less), allowing adaptations fuelled by multiple interacting genes to evolve [67]. In addition, our results highlight the efficacy of the classical formulation of Hamilton's rule for explaining cooperation in human populations.

We have shown that cooperation is predominantly, though not exclusively, structured around kinship relations in a rural farming community in southwest China. Thus, our results speak to the importance of understanding human cooperation through the lens of inclusive fitness.

## Footnotes

1 The concept of tolerated scrounging includes transfers due to relative need and spatial proximity, as well as an inability to monopolize resources due to pressure from solicitation or dominant individuals [20]. Here, we are concerned with the effects of need and proximity, and separate them in our analyses rather than lumping them under a single umbrella term.

## Data accessibility

Anonymized data are available from Dryad http://dx.doi.org/10.5061/dryad.q24m6 [68]; note that due to privacy concerns, we are unable to make all covariates publicly accessible. R code to reproduce the analyses is available from https://github.com/matthewgthomas/mosuo-kinship.
